# Interactions of Zn(II) Ions with Three His-Containing Peptide Models of Histone H2A

**DOI:** 10.1155/S1565363304000093

**Published:** 2004

**Authors:** Marios Mylonas, Artur Krężel, John C. Plakatouras, Nick Hadjiliadis, Wojciech Bal

**Affiliations:** 1 University of Ioannina Department of Chemistry Ioannina 45110 Greece; 2 Faculty of Chemistry University of Wroclaw Wroclaw 50-383 Poland; 3 Institute of Biochemistry and Biophysics Polish Academy of Sciences Warsaw 02-106 Poland

## Abstract

The interactions of Zn(ll) ions with the blocked hexapeptide models -TESHHK-, -TASHHK- and -TEAHHK- of the -ESHH- motif of the C-terminal of historic H2A were studied by using potentiometric and IH-NMR techniques. The first step of these studies was to compare the pKa values of the two His residues inside each hexapeptide calculated by potentiometric or H-NMR titrations. Hereafter, the potentiometric titrations in the pH range 5 11 suggest the formation of several monomeric Zn(ll) complexes. It was found that all hexapeptides bind to Zn(ll) ions initially through both imidazole nitrogens in weakly acidic and neutral solutions forming slightly distorted octahedral complexes. At higher pH values, the combination of potentiometric titrations and one and two dimensional NMR suggested no amide coordination in the coordination sphere of Zn(II) ions. Obviously, these studies support that the -ESHH- sequence of histone H2A is a potential binding site for Zn(II) ions similarly with the Cu(II) and Ni(ll) ions, presented in previous papers.


### Interactions of Zn(II) Ions with Three His-Containing Peptide Models of Histone H2A

Marios Mylonas,[a] Artur Krężel,[b] John C. Plakatouras,*[,a] Nick Hadjiliadis,*[,a] and Wojciech Bal[c]

[a] *University of Ioannina, Department of Chemistry, Ioannina 45110, Greece*
[b] *Faculty of Chemistry, University of Wroclaw, 50-383 Wroclaw, Poland*
[c] *Institute of Biochemistry and Biophysics, Polish Academy of Sciences, 02-106 Warsaw, Poland*

### ABSTRACT

The interactions of Zn(II) ions with the blocked hexapeptide models -TESHHK-, -TASHHK- and -TEAHHK- of the -ESHH- motif of the C-terminal of histone H2A were studied by using potentiometric and $^1$H-NMR techniques. The first step of these studies was to compare the p$K_a$ values of the two His residues inside each hexapeptide calculated by potentiometric or $^1$H-NMR titrations. Hereafter, the potentiometric titrations in the pH range 5 - 11 suggest the formation of several monomeric Zn(II) complexes. It was found that all hexapeptides bind to Zn(II) ions initially through both imidazole nitrogens in weakly acidic and neutral solutions forming slightly distorted octahedral complexes. At higher pH values, the combination of potentiometric titrations and one and two dimensional NMR suggested no amide coordination in the coordination sphere of Zn(II) ions. Obviously, these studies support that the -ESHH- sequence of histone H2A is a potential binding site for Zn(II) ions similarly with the Cu(II) and Ni(II) ions, presented in previous papers.

### INTRODUCTION

It is well known that several metals have been found to be carcinogenic to humans and animals /1,2/. Nevertheless the molecular mechanism by which metal carcinogenicity is exerted is not fully understood. Studies of several years support that neoplastic transformation of cells results from a heritable alteration in the genetic code, concluding that any molecule that can bind with constituents of the cell nuclei may affect the genetic code. Thus, it is believed that metal ions may cause changes to the genetic code by their binding to the proteins and DNA. Especially, the abundance of histones makes them the prime expectants for that role. It must be noticed that the nucleosomes represent the first level of DNA compaction in eukaryotic nuclei and consist of 146 bp of core DNA wrapped around a cluster of eight histone proteins (containing two copies of each of histones H2A, H2B, H3 and H4), with 20-60 bp of linker DNA joining adjacent cores /3/.

Obviously, the studies of metal interactions with the peptides or protein fragments, especially histones, can lead in explaining mechanisms related to cancer and toxicity caused by metals. As a consequence, the coordination properties of Ni(II) ions towards the tetrapeptide Ac-CysAlaIleHis-am (-CAIH-) representing the 110-113 residues of histone H3 /4-6/ and the peptide model Ac-AlaLysArgHisArgLys-am (-AKRHRK-) of the N-terminal "tail" of histone H4 and its modifications has been reported /7,8/. In the case of the peptide model of histone H3, it was established that Ni(II) ions form a very strong complex, that could mediate oxidative damage to DNA /4-6/. In addition, it was found that at pH above 7, Ni(II) complexation with the hexapeptide Ac-ThrGluSerHisHisLys-am (-TESHHK-) and bigger peptide models containing the C-terminal "tail" -ESHH-, which corresponds to the 121-124 residues of a major variant of mammalian histone H2A, was accompanied by hydrolytic cleavage of the -Glu-Ser- peptide bond and the formation of a square-planar Ni(II) complex with the resulting -SHHK- sequence /9,10/. It must be noticed that Cu(II) ions provide similar hydrolytic activity with kinetics more than three times slower than those for Ni(II) /10/.

Furthermore, we studied the interactions of Cu(II) ions with the same H2A histone hexapeptide model -TESHHK- /11/ and compared the results to those of Ni(II) ions /9/. The Cu(II)-TESHHK- complex which was formed at about pH 7.4 was able to induce oxidative damage of 2′-deoxyguanosine (dG), in the presence of hydrogen peroxide /11/. Later, a systematic study of interactions of Ni(II) and Cu(II) ions with the hexapeptides Ac-ThrAlaSerHisHisLys-am (-TASHHK-), Ac-ThrGluAlaHisHisLys-am (-TEAHHK-), Ac-ThrGluSerAlaHisLys-am (-TESAHK-) and Ac-ThrGluSerHisAlaLys-am (-TESHAK-), peptide models of the -ESHH- motif of histone H2A, led us to conclude that these sequences are potential binding sites for Ni(II) and Cu(II) ions and additionally the presence of Ser and His-5 residues inside the peptide sequence may be critical for the hydrolysis reaction caused by Ni(II) or Cu(II) ions /11-14/.

Bearing in mind that except Ni(II) and Cu(II) ions /15,16/, His residues are the major coordination sites in the active centers of several Zn(II)-containing enzymes /17-19/, in this paper we decided to study the interactions of the blocked hexapeptides -TESHHK-, -TASHHK- and -TEAHHK-, peptide models of the -ESHH- motif of histone H2A, with Zn(II) ions. We chose only the hexapeptides containing two His residues from the five hexapeptides we have studied previously /11-14/, because in most of the active centers of Zn(II)-containing enzymes, Zn(II) ions are bound in more than one His residue /17-19/. The next step of this work will be the study of the possibility of Zn(II) ions to cleave the -ESHH- motif of histone H2A, similarly with Ni(II) and Cu(II) ions /11-14/.

### EXPERIMENTAL

### Materials

$Zn(NO_3)_2{\cdot}4H_2O$, $HNO_3$, $KNO_3$, acetonitrile (HPLC grade), NaOH and dicyclohexylcarbodiimide (DCC) were obtained from E. Merck (Darmstadt, Germany). 1-hydroxybenzotriazole (1-HOBt), trifluoroacetic acid (TFA), trifluoroethanol (TFE), 3-(trimethylsilyl)propionic acid sodium salt (TSP), anisole, $D_2O$ and DCl were purchased from Aldrich Chemical Co. (Milwaukee, WI). Isopropanol, dimethylformamide, diethylether and dichloromethane were purchased (analytical grade) from Lab-Scan Chemical Co. (Dublin, Ireland). The protected amino acids, Fmoc-His(Mtt)-OH, Fmoc-Lys(Boc)-OH, Fmoc-Ser(tBu)-OH, Fmoc-Glu(tBu)-OH, Fmoc-Thr(tBu)-OH and Fmoc-Ala-OH and the resin H-Linker-2-chlorotrityl were purchased from CBL Chemicals Ltd. (Patras, Greece).

### Peptide synthesis

The blocked hexapeptides -TESHHK-, -TASHHK- and -TEAHHK- were synthesized in the solid phase, as described previously /12,13/. ESI-MS and $^1$H-NMR techniques were used for the characterization of the peptides /12,13/.

### Potentiometry

The protonation and stability constants of Zn(II) complexes of -TESHHK-, -TASHHK- and -TEAHHK-, in the presence of 0.1 M $KNO_3$, were determined by using pH-metric titrations over the pH range 2.5 - 11, at 25 °C, with 0.1 M NaOH as titrant (Molspin automatic titrator, Molspin Ltd., Newcastle-upon-Tyne, U.K.). Changes of pH were monitored with a combined glass-silver chloride electrode calibrated daily in $H^+$ concentrations by $HNO_3$ titrations /20/. The time to reach pH-equilibrium during titrations varied from 1-10 min, depending on the pH value. Sample volumes of 1.5 mL and concentrations of 1 mM of the hexapeptides and 0.5 – 1 mM $Zn(NO_3)_2{\cdot}4H_2O$ were used. The experimental data were analyzed using the SUPERQUAD program /21/. Standard deviations computed by SUPERQUAD refer to random errors.

### NMR spectroscopy

$^1$H-NMR experiments were performed on a Bruker AMX 400MHz spectrometer. The one dimensional experiments were carried out in 1:4 $D_2O$:$H_2O$ mixtures at a peptide concentration of 5 mM of both free and Zn(II)-bound hexapeptide -TASHHK-, in peptide - Zn(II) ratio 1.2:1 at pH$^*$ 10.5 and 25 °C (The pH$^*$ reading of the electrode was not corrected for the isotope effect). TOCSY experiments was used to assign the spectra of both free and Zn(II)-bound hexapeptide -TASHHK-, in peptide - Zn(II) ratio 1.2:1 ($c_L$ = 15 mM) in the same pH$^*$ and temperature. Finally, $^1$H-NMR titrations were carried out for the hexapeptides -TESHHK-, -TASHHK- and -TEAHHK- (c = 5 mM) in 99.9 % $D_2O$ solutions covering the pH$^*$ range 2 - 11 at 25 °C, used to calculate the p$K_a$ values of $N_3$ nitrogen atoms of imidazole rings of the two His residues.

### RESULTS AND DISCUSSION

### Acid-base behavior of free hexapeptides

Both the N- and C-terminal of the hexapeptides models (Scheme 1) were blocked by acetylation and amidation, respectively, to make the peptides more realistic models of the -ESHH- motif of histone H2A. As has already been written in the Experimental Section, ESI-MS and $^1$H-NMR techniques were used for the characterization of the hexapeptides but the data have been already reported in previous papers /12,13/.

The first two hexapeptides -TESHHK-, -TEAHHK- and the hexapeptide -TASHHK- contain four and three groups, respectively, which are capable of reversible proton binding. These groups are the carboxyl group of Glu residue, the $N_3$ imidazole nitrogens of His residues and the ε-amino group of Lys residue. The protonation constants and dissociation macroconstants of these groups were measured by potentiometric titrations and are presented in Table 1. The highest p$K_a$ values between 10.48 - 10.28 can be easily assigned to the ε-amino group of Lys residues and the lowest values at 3.85 and 4.10 of -TESHHK- and -TEAHHK-, respectively, can be assigned to the carboxyl group of Glu residues, according to the literature data /9,22,23/.

**Table 1**

Protonation constants (log $\beta$) and dissociation macroconstants (p$K_a$) of the hexapeptides -TESHHK-, -TASHHK- and -TEAHHK- (T = 25 °C, I = 0.1 M $KNO_3$)

| | $H_4L$ | | $H_3L$ | | $H_2L$ | | HL | |
|---|---|---|---|---|---|---|---|---|
| | log $\beta^*$ | p$K_{a1}$ | log $\beta^*$ | p$K_{a2}$ | log $\beta^*$ | p$K_{a3}$ | log $\beta^*$ | p$K_{a4}$ |
| **-TESHHK-** | 26.81 (1) | 3.85 | 22.96 (1) | 5.90 | 17.06 (1) | 6.78 | 10.28 (1) | 10.28 |
| **-TASHHK-** | — | | 22.85 (1) | 5.74 | 17.10 (1) | 6.62 | 10.48 (1) | 10.48 |
| **-TEAHHK-** | 27.03 (1) | 4.10 | 22.93 (1) | 6.00 | 16.93 (1) | 6.68 | 10.25 (1) | 10.25 |

* standard deviations of the last digit are given in parentheses

**Scheme 1**

Full protonated species of the used peptides.

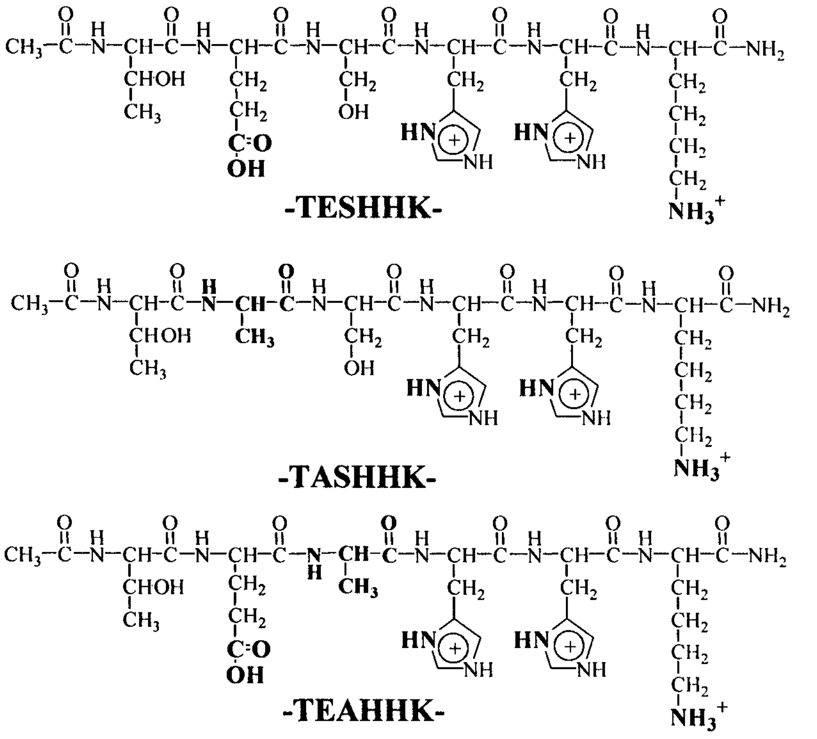

The other values of macroconstants corresponding to the deprotonations of His residues were found to be separated by less than one log unit, suggesting a possibility of concurrent deprotonations at the two His residues. The comparison with the literature indicates that the lowest p$K_a$ value should mainly correspond to the protonation of the His residue closer to the N-termini of the peptide, while the closest His in the C-terminal has a higher basicity /24-26/.

**Table 2**

Dissociation macroconstants (p$K_a$) of the hexapeptides -TESHHK-, -TASHHK- and -TEAHHK- calculated on the basis of potentiometric and spectroscopic ($^1$H-NMR) data

| | -TESHHK- | | -TASHHK- | | -TEAHHK- | |
|---|---|---|---|---|---|---|
| | p$K_a$ | | p$K_a$ | | p$K_a$ | |
| | His-4 | His-5 | His-4 | His-5 | His-4 | His-5 |
| **Potentiometry** | **5.90** | **6.78** | **5.74** | **6.62** | **6.00** | **6.68** |
| **Henderson-Hasselbalch method** [a] | 6.35 (1) | 6.41 (2) | 6.29 (2) | 6.30 (2) | 6.42 (2) | 6.48 (2) |
| **Rabenstein-Sayer method** [b] | 6.12 (4) | 6.88 (5) | 6.06 (3) | 6.61 (4) | 6.15 (3) | 6.72 (2) |

* standard deviations of the last digit are given in parentheses, [a] ref. 27 and 28, [b] ref. 29

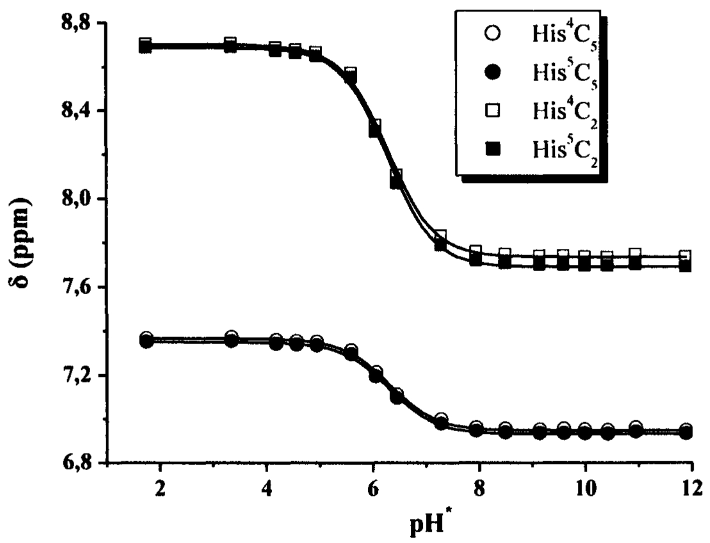

**Fig. 1:** Chemical shifts of the imidazole protons of $C_2$ or $C_5$ carbon atoms of hexapeptide -TASHHK- as a function of pH*. Henderson-Hasselbalch method was used for the fitting of the data.

Obviously, the two His residues in these hexapeptides have multiple acid-base equilibria. It is known that potentiometric titrations cannot always resolve these overlapping proton dissociations. On the contrary, NMR spectroscopy investigates individual protons in peptides or proteins, facilitating the study of specific proton association-dissociation equilibrium in polyprotic systems. Thus, a series of one dimensional $^1$H-NMR

spectra of all hexapeptides at various pH* values were recorded for the further verification of the p$K_a$ values of the two His residues, calculated from the potentiometric data. Generally, it is known that the proton dissociation can be easily monitored from the chemical shifts of the neighboring protons at each pH* value. In particular, the dissociation of the $N_3$ imidazole nitrogen atoms can be calculated from the plot of chemical shifts of the adjacent protons of $C_2$ and $C_5$ carbon atoms of imidazole rings as a function of pH*.

It must be mentioned that the calculation of these p$K_a$ values was realized using two different methods. In the first one, the p$K_a$ values of the two His residues were calculated independently /27,28/. In contrast, in the second method the two p$K_a$ values calculated together /29/.

In Figure 2 an example plot produced from the chemical shifts of the imidazole protons of $C_2$ of hexapeptide –TASHHK– is presented. Similar plots were produced for all hexapeptides.

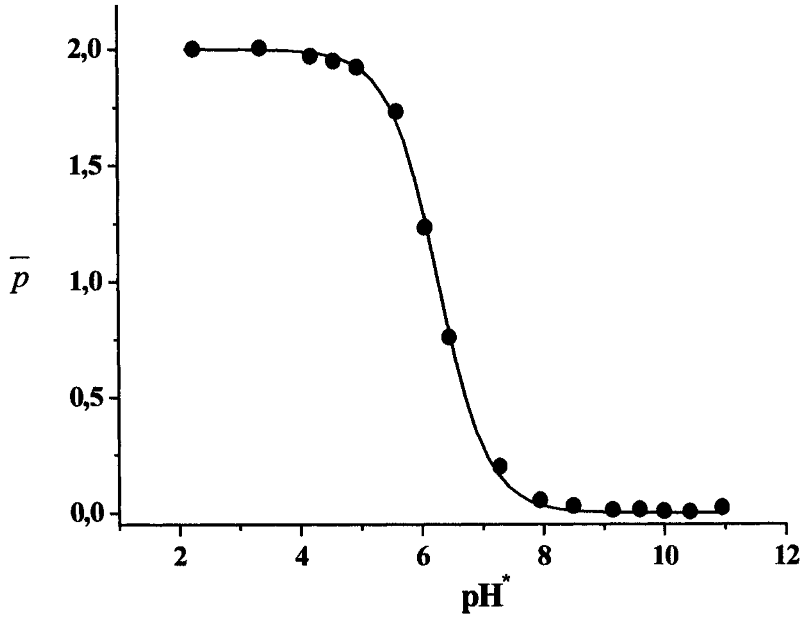

**Fig. 2:** Plot of factor $\bar{p}$ as a function of pH*. The parameter $\bar{p}$ presents the number of protons per acid molecule and it has been calculated using the chemical shifts of the imidazole protons of $C_2$ carbon atoms of both His residues, in the case of the hexapeptide -TASHHK-. Rabenstein-Sayer method was used for the fitting of the data.

Obviously, the p$K_a$ values of two His residues of all hexapeptides calculated using the first method are not equivalent with the comparable values calculated from the potentiometric titrations (Table 2). Thereby, the p$K_a$ values of two neighboring groups as it is occurred in the case of the hexapeptides -TESHHK-, -TASHHK- and -TEAHHK- cannot be extracted with rewarding accuracy using the first method. In contrast, the same p$K_a$ values calculated using the second method are similar with the comparable values calculated from the potentiometric titrations (Table 2). Additionally, the use of the second method led to more realistic and accurate values because for the calculations we took into account the presence of both His residues.

### Zinc Complexation

Potentiometric titrations in aqueous solutions were carried out for the hexapeptides in the presence of zinc nitrate in peptide : Zn(II) ratios 1:1 and 2:1. The stability constants of Zn(II) complexes, calculated from these titrations using the SUPERQUAD program are presented in Table 3. The species distribution diagrams presented in Figure 3 indicate the formation of four complexes (ZnHL, ZnL, $ZnH_{-1}L$ and $ZnH_{-2}L$) in the case of Zn(II) / -TESHHK- and Zn(II) / -TEAHHK- systems and two complexes (ZnHL and $ZnH_{-2}L$) in the case of Zn(II) / -TASHHK- system. Binuclear complexes of type $Zn_2L_x$ or complexes with two ligands were repeatedly rejected by SUPERQUAD program /21/ and were eliminated from the model.

**Table 3**

Stability constants (log $\beta$) of Zn(II) complexes with the hexapeptides

-TESHHK-, -TASHHK- and -TEAHHK- (T = 25 °C, I = 0.1 M $KNO_3$)

| | ZnHL | ZnL | | $ZnH_{-1}L$ | | $ZnH_{-2}L$ | |
|---|---|---|---|---|---|---|---|
| | log $\beta^*$ | log $\beta^*$ | $pK_{a1}$ | log $\beta^*$ | $pK_{a2}$ | log $\beta^*$ | $pK_{a3}$ |
| **-TESHHK-** | 13.45 (2) | 5.92 (2) | 7.53 | -2.05 (2) | 7.97 | -12.35 (8) | 10.30 |
| **-TASHHK-** | 13.39 (2) | — | | — | | -11.08 (2) | |
| **-TEAHHK-** | 13.70 (2) | 6.59 (1) | 7.11 | -2.07 (5) | 8.66 | -12.41 (3) | 10.34 |
| **-HH-**[33] | 10.05 | 4.19 | | | | | |
| **c-HH**[35] | | 2.55 | | | | | |
| **c-GH**[35] | | 1.71 | | | | | |
| **GH**[30] | 10.87 | 3.98 | | -2.75 | | -12.66 | |
| **AH**[31] | 10.30 | 3.50 | | -3.60 | | | |
| **GHist**[32] | 9.91 | 3.58 | | | | | |
| **SarHist**[32] | 10.01 | 3.08 | | | | | |
| **-HPH-**[34] | 9.41 | 3.29 | | | | | |
| **HmSH**[38] | | | | -3.13 | | -12.87 | |
| **GHK**[34] | | | | -2.30 | | -11.80 | |
| **GHG**[35] | | | | -2.55 | | -12.24 | |

* $\beta = /Zn_iH_jL_k/ / (/Zn/^i /H/^j /L/^k)$, standard deviations of the last digit are given in parentheses
Hist = histamine, Sar = sarcosyl, HmS = α-hydroxymethylserine

As can be seen from Figure 3, the coordination of all hexapeptides starts in pH above 5 and the ZnHL complexes are formed. The higher values of their stability constants (Table 3) comparing with the analogues complexes with GlyHis /30/, AlaHis /31/, GlyHist /32/, SarHist /32/ and with protected peptides -HisHis- /33/ and -HisProHis- /34/ support the coordination of all hexapeptides through both imidazole rings. It must be mentioned that for the ZnHL complexes with the above dipeptides and the protected peptides the imidazole or amino monodentate binding /30-34/ have been suggested. Additionally, the carboxylate oxygen of Glu residue of -TESHHK- and -TEAHHK-, which is deprotonated in the pH range of the formation of these complexes, may also participate in the coordination sphere of Zn(II) ions forming a slightly distorted octahedral complexes similarly with the analogues Ni(II) and Cu(II) complexes with the same hexapeptides /11-13/.

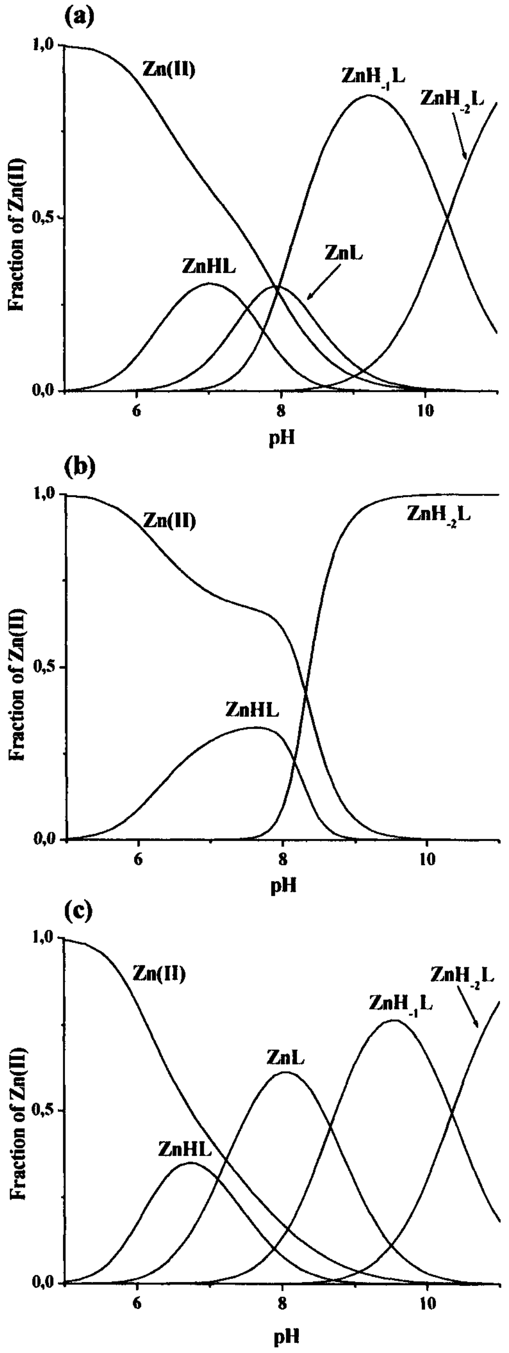

**Fig. 3:** Species distribution diagrams of Zn(II) complexes with (a) -TESHHK-, (b) -TASHHK- and (c) -TEAHHK- ($c_{Zn} = c_L$ = 1 mM, T = 25 °C, I = 0.1 M $KNO_3$).

Increasing the pH, the complexes ZnL and $ZnH_{-1}L$ with hexapeptides -TESHHK- and -TEAHHK- release additional protons with $pK_a$ 7.53 and 7.11 (ZnL), 7.97 and 8.66 ($ZnH_{-1}L$), respectively (Table 3). Although these $pK_a$ values are comparable with the $pK_a$ values belonging to His-containing unprotected peptides with the amide coordination and could be abstracted from amide nitrogens /30,31,36-38/, we do not support the amide coordination of both hexapeptides. Generally, it is known that amide deprotonations is more difficult to observe with Zn(II) ions than other metal ions, including Cu(II) and Ni(II) ions /16/. At high pH values it is possible to observe this type of coordination but additionally competition from hydroxo complex formation is limitative. Thus, Zn(II) ions exhibit a stronger tendency to undergo hydrolysis than amide coordination. Concluding, the most probable hypothesis about the two deprotonations leading to the formation of ZnHL → ZnL → $ZnH_{-1}L$ complexes may be the successive deprotonation of already bound water molecules. This is in agreement with the preliminary analysis of the titration data which indicated titration of two more protons in the case of the hexapeptide / Zn(II) systems than in the case of the free hexapeptides.

As can be seen in Table 3, the stability constants of ZnL complexes with the hexapeptides -TESHHK- and -TEAHHK- are higher than that of ZnL complexes with the protected dipeptide -HisHis- (log $\beta$ = 4.19) /33/ and with the cyclic dipeptide c-HisHis (log $\beta$ = 2.55) /35/, in which the coordination of both imidazole rings to the metal ions were proposed. Obviously, ZnL complexes with the studied hexapeptides are considerably more stable than the corresponding complexes of the above reported and other His-containing peptides which are coordinated through one or two nitrogen atoms (Table 3). The higher stability of the studied complexes may contribute from the carboxyl group of Glu residue which remained bound to the metal ions. It is worthy to note that the absence of Ser residue stabilize the ZnL complex with -TEAHHK- comparing with the corresponding complex with -TESHHK- (Table 3).

In contrast, the stability constants of $ZnH_{-1}L$ complexes with the hexapeptides -TESHHK- and -TEAHHK- are similar to the related complexes with GlyHis /30/, AlaHis /31/, GlyHisLys /34/, GlyHisGly /35/ and HmSHis /38/ which all correspond to the {$NH_2$, $N^-$, $N_{Im}$} donor set involved in the equatorial plane of Zn(II) ions (Table 3). Although amide coordination in $ZnH_{-1}L$ complexes with the hexapeptides -TESHHK- and -TEAHHK- is not suggested, the participation of the carboxyl group of Glu residue and the two imidazole rings in coordination sphere of Zn(II) ions may result in similar stabilization with the {$NH_2$, $N^-$, $N_{Im}$} mode.

Finally, above pH 8 the deprotonation of the $ZnH_{-1}L$ complexes with the hexapeptides -TESHHK- and -TEAHHK- takes place with a $pK_a$ 10.30 and 10.34, respectively, forming the $ZnH_{-2}L$ complexes (Table 3). These $pK_a$ values are in good agreement to that for protonation of ε-amino group of Lys residue, $pK_\alpha$ 10.28 and 10.25 (Table 1), in free ligands -TESHHK- and -TEAHHK-, respectively. Obviously, the $ZnH_{-2}L$ complexes provide a similar coordination mode with $ZnH_{-1}L$ complexes, differing to the deprotonated and uncoordinated ε-amino group of Lys residue similarly with the analogues Cu(II) and Ni(II) complexes with the studied hexapeptides /11-13/.

Fitting of the titration data of the Zn(II) / -TASHHK- system can be done only considering the ZnHL and $ZnH_{-2}L$ species. This may be due to simultaneous deprotonation of the coordinated water protons and the protonated ε-amino group of Lys.

The proposed structures of some selected species are presented in Figure 4.

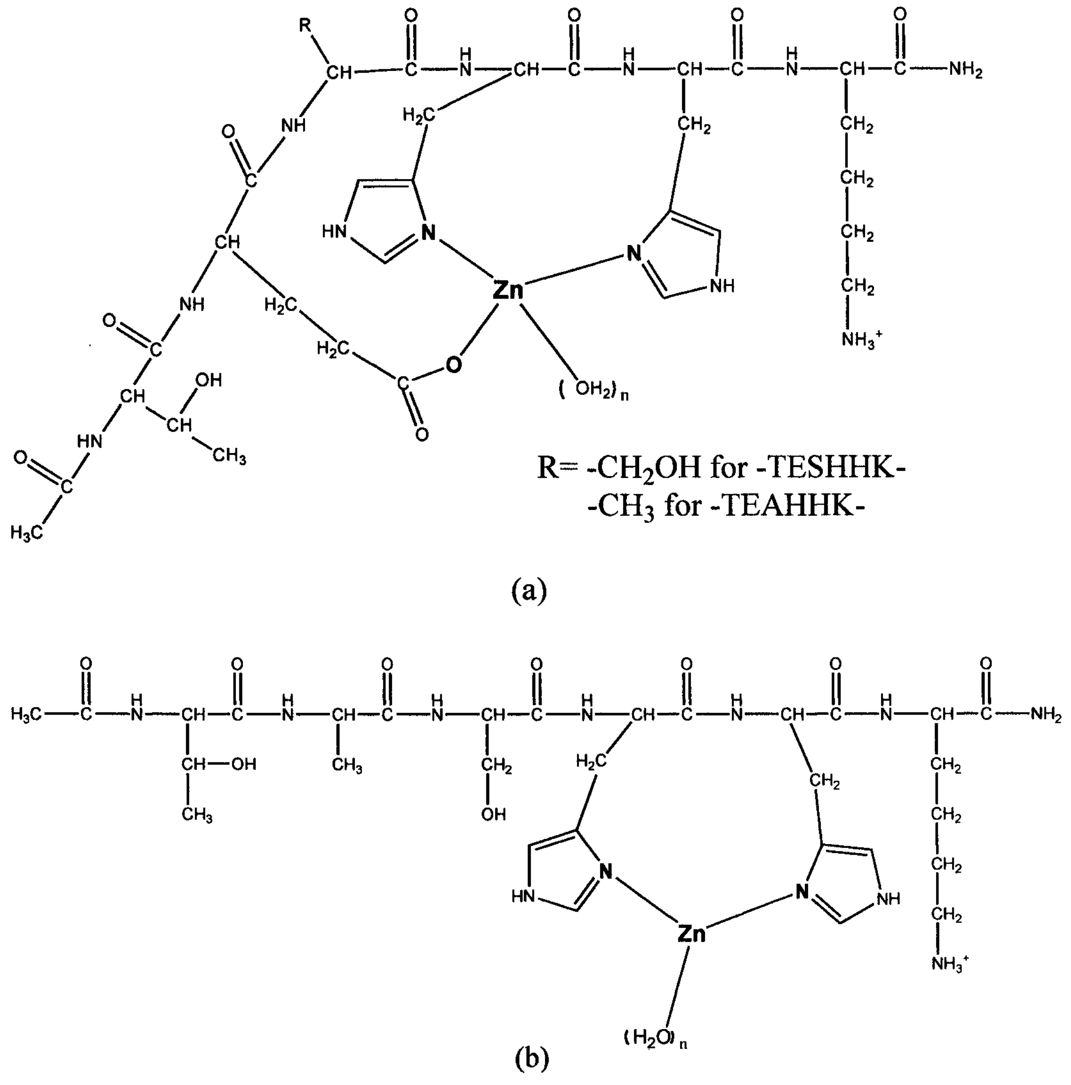

**Fig. 4:** The proposed structures for the species ZnHL. (a) –TESHHK– and –TEAHHK–, (b) –TASHHK–, n = 2 – 4.

In order to study further the proposed structures we decided to use NMR spectroscopy. Bearing in mind that the coordination sphere of all species with all hexapeptides is similar we tried to study ZnHL and $ZnH_{-2}L$ complexes. We chose Zn(II) / -TASHHK- system for these NMR studies because the above complexes exists without overlaps at their formation pH range only in the case of the hexapeptide -TASHHK- (Figure 3). Firstly, $^1$H-NMR spectra of -TASHHK- were recorded, at pH$^*$ 7.3 and 25 °C, in the absence and presence of Zn(II) ions (ratio 1:1, in $D_2O$:$H_2O$ 1:4 mixture). Unfortunately, the spectrum in the presence of Zn(II) was not helpful due to the extensive broadening of all signals which was derived from the high concentration of free metal ions (Figure 3). Thus, $^1$H-NMR (Figure 5) and TOCSY (Figure 6) spectra of -TASHHK- at pH$^*$ 10.30 were recorded, in the absence and presence of Zn(II) ions (peptide - Zn(II) ratio 1.2:1, in $D_2O$:$H_2O$ 1:4

mixture). The chemical shifts of $^{1}H$ (δ, ppm) of free and bound -TASHHK- at pH* 10.30 are presented in Table 4.

**Table 4**
$^{1}$H-NMR assignment of -TASHHK-, in absence or presence of Zn(II) ions in peptide - Zn(II) ratio 1.2:1 at pH* 10.3 (ppm relative to TSP)

| | -TASHHK- | | |
|---|---|---|---|
| | **Free** | **Bound** | **Δδ** |
| *acetyl* | 2.13 | 2.17 | +0.04 |
| *Thr α* | 4.27 | 4.32 | +0.05 |
| *β* | 4.25 | 4.28 | +0.03 |
| *γ* | 1.27 | 1.27 | 0.00 |
| *Ala α* | 4.42 | 4.44 | +0.02 |
| *β* | 1.42 | 1.45 | +0.03 |
| *Ser α* | 4.41 | 4.46 | +0.05 |
| *β'* | 3.84 | 3.87 | +0.03 |
| *β''* | 3.88 | 3.94 | +0.06 |
| *His⁴ α* | 4.62 | 4.66 | +0.04 |
| *β* | 3.09 | 3.12 | +0.03 |
| $C_2$ | 7.73 | 7.24 | -0.49 |
| $C_5$ | 6.94 | 6.98 | +0.04 |
| *His⁵ α* | 4.60 | 4.62 | +0.02 |
| *β* | 3.08 | 3.08 | 0.00 |
| $C_2$ | 7.68 | 7.19 | -0.49 |
| $C_5$ | 6.91 | 6.95 | +0.04 |
| *Lys α* | 4.30 | 4.29 | -0.01 |
| *β'* | 1.78 | 1.80 | +0.02 |
| *β''* | 1.89 | 1.89 | 0.00 |
| *γ* | 1.48 | 1.50 | +0.02 |
| *δ* | 1.73 | 1.70 | -0.03 |
| *ε* | 2.98 | 3.01 | +0.03 |

It is well known that the complexation of several peptides to the metal ions produces significant chemical shift changes of the signal of the protons near the binding sites in NMR spectra due to the electron density shift to the metal ions. Obviously, the comparisons of $^{1}$H-NMR and TOCSY spectra between free and bound -TASHHK- (Table 4) indicated that the positions of the protons of Thr, Ala, Ser and Lys residues were almost not affected, suggesting that they were not involved in the coordination sphere of Zn(II) ions. It must be noticed that remarkable chemical shift changes of the signals belonging to α proton were not observed, suggesting the absence of bound amide nitrogen from the coordination sphere of Zn(II) ions.

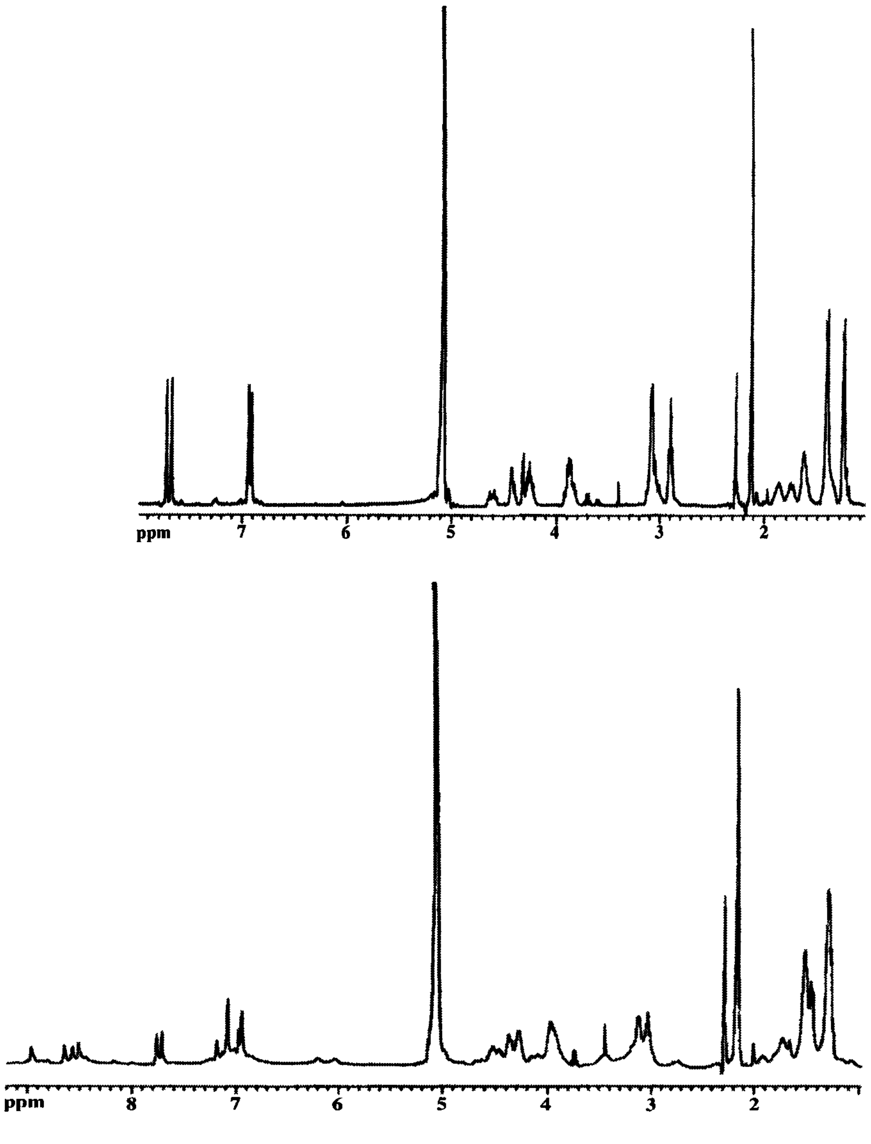

**Fig. 5:** $^1$H-NMR spectra of -TASHHK- in the absence (up) or presence (down) of Zn(II) ions, in peptide - Zn(II) ratio 1.2: 1 ($c_L$ = 5 mM) and pH* 10.3, in $D_2O$:$H_2O$ 1:4 mixture.

In the imidazole protons region of $^1$H-NMR spectrum of the -TASHHK- in the presence of Zn(II) ions, new peaks were observed comparing with the corresponding spectrum in the absence of Zn(II) ions and supporting the complexation of Zn(II) ions through the imidazole rings of His residues (Figure 5). Most of them observed above 8.00 ppm may easily correspond to the signals of the peptide hydrogens (-NH-). In contrast we must report that signals of the peptide hydrogens in the $^1$H-NMR spectrum of free -TASHHK- were not detected. Additionally, it was found that the two new peaks below 8.00 ppm, and two peaks which are overlapping with the already existing peaks of free hexapeptide, produced two new cross-peaks in the TOCSY experiment [Im $C_2$-H / Im $C_5$-H: 7.24 / 6.98 ppm and 7.19 / 6.95 ppm] (Figure 6) corresponding to the two His residues in bound hexapeptide. The differences and also the similarity of them in chemical shifts of the signals of the imidazole protons observed in both His residues (Table 4), in the presence of Zn(II) ions, clearly indicate the participation of both imidazole rings in coordination sphere of Zn(II) ions. Finally, it must be mentioned that the chemical shifts of the imidazole protons of free hexapeptide (with negligible changes) were also detected in the same region of $^1$H-NMR spectrum [$Im^4$ $C_2$-H / $Im^4$ $C_5$-H: 7.76 ppm / 6.98 ppm and $Im^5$ $C_2$-H / $Im^5$ $C_5$-H: 7.71 ppm / 6.95 ppm] due to the small excess of ligand we used to achieve shorter complex formation equilibrium times during the NMR experiments.

Concluding, without exception, the NMR data presented in this paper suggest the same coordination mode of $ZnH_{-2}L$ complexes $\{2N_{Im}\}$ with the hexapeptide -TASHHK- as has also been supported by potentiometric measurements of the analogues complexes with all hexapeptides.

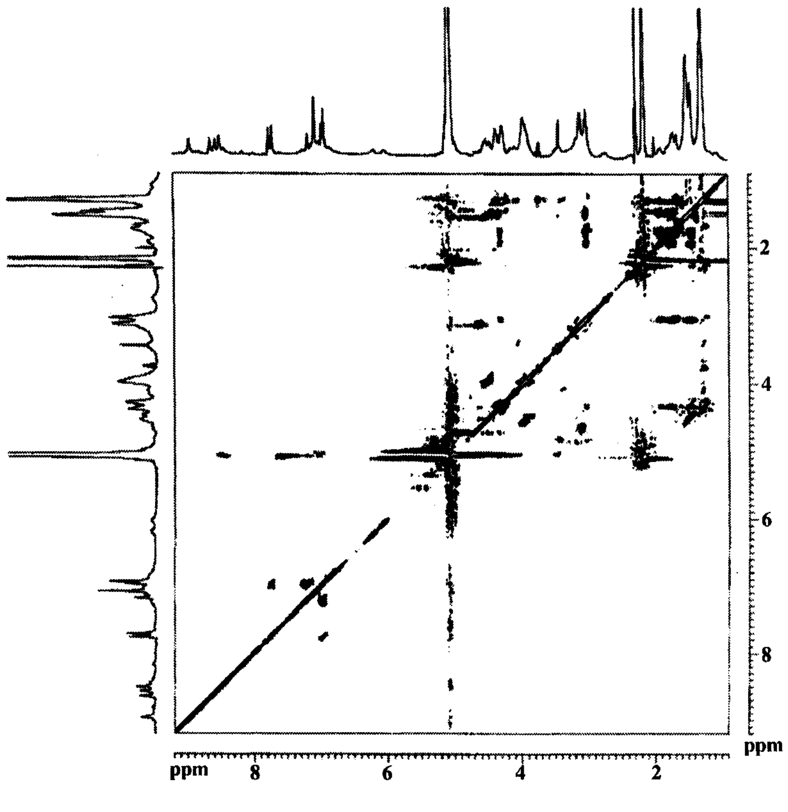

**Fig. 6:** TOCSY spectrum of -TASHHK- in presence of Zn(II) ions, in peptide - Zn(II) ratio 1.2:1 ($c_L$ = 15 mM) and pH* 10.3, in $D_2O$:$H_2O$ 1:4 mixture.

### CONCLUSIONS

The studies with the blocked hexapeptide models -TESHHK-, -TASHHK- and -TEAHHK- of the -ESHH- motif of the C-terminal of histone H2A, presented in this paper, support that this sequence is a potential binding site for Zn(II) ions similarly with the Cu(II) and Ni(II) ions /11-13/.

Firstly, a combined use of potentiometric and $^1$H-NMR titrations has allowed us to compare the macroconstants in protonation equilibria of the two His residues inside the sequence of the studied hexapeptides. Afterwards the potentiometric titrations in aqueous solutions of hexapeptide / Zn(II) systems, indicated that several monomer Zn(II) complexes are formed in the pH range 5 - 11. It was found that these complexes were apparently less stable than the corresponding Cu(II) complexes with the same hexapeptides /11,12/. Besides it is well known that Zn(II) complexes are kinetically labile, leading to several equilibria between complexes with different donor sets and distorted geometries or between coordinated and uncoordinated forms /39/. The potentiometric data suggested the initial coordination of all hexapeptides through both imidazole rings and additionally through the carboxylate oxygen of Glu residue in the case of -TESHHK- and -TEAHHK-, forming a slightly distorted octahedral complexes similarly with the analogues Ni(II) and Cu(II) complexes with the same hexapeptide models /11-13/. In more basic solutions, the most probable interpretation of the predominated complexes is the deprotonation of bound water molecules and ε-amino group of Lys residue. The last proposed structures of complexes existing in basic pH values, have been studied also by one and two (TOCSY) dimensional NMR techniques leading to the same coordination features.

Obviously, binding of Zn(II) ions to the C- terminal of histone H2A may inevitably change its conformation, disturbing the interactions of histone H2A inside the histone octamer with the other histones, DNA and other molecules. Additionally, it is well known that Zn(II) ions are able to bind with several molecules inside the cells, including reduced glutathione (GSH) which is one of the most abundant molecules of life (c = 1 - 10 mM intracellularly) /40/ and free histidine which also exists in high concentrations (c = 0.1 mM) and it has been proposed as a carrier of Zn(II) ions in some tissues /41/. These observations clearly indicate the great biological interest relative to the binding of Zn(II) ions inside the cells. Thus, the next step in our studies needs to ascertain the ability of Zn(II) ions to catalyze the hydrolysis of the studied hexapeptides in physiological conditions, similarly with Ni(II) and Cu(II) ions /9,13,14/.

### REFERENCES

1. K. S. Kasprzak in *"Cytotoxic, Mutagenic and Carcinogenic Potential of Heavy Metals Related to Human Environment"*, NATO ASI Ser. 2, (ed. N. Hadjiliadis), Environment, Kluwer, Dordrecht, 26 (1997) 73.
2. K. S. Kasprzak. Oxidative DNA damage in metal-induced carcinogenesis. In: *Toxicology of metals*, L. W. Chang, Ed., vol 18 (1996).
3. K. Luger, A. W. Mader, R. K. Richmond, D. F. Sargent and T. J. Richmond. *Nature*. **389** 251 (1997).

4. W. Bal, J. Lukszo, M. Jeżowska-Bojczuk and K. S. Kasprzak. *Chem. Res. Toxicol.* **8**, 683 (1995).
5. W. Bal and K. S. Kasprzak in *"Cytotoxic, Mutagenic and Carcinogenic Potential of Heavy Metals Related to Human Environment"*, NATO ASI Ser. 2, (ed. N. Hadjiliadis), Environment, Kluwer, Dordrecht, 26 (1997) 107.
6. W. Bal, J. Lukszo and K. S. Kasprzak. *Chem. Res. Toxicol.* **9**, 535 (1996).
7. M. A. Zoroddu, T. Kowalik-Jankowska, H. Kozlowski, H. Molinari, K. Salnikow, L. Broday and M. Costa. *Bioch. Biophy. Acta.* **1475**, 163 (2000).
8. M. A. Zoroddu, M. Peana, T. Kowalik-Jankowska, H. Kozlowski and M. Costa. *J. Chem. Soc., Dalton Trans.* **2002**, 458.
9. W. Bal, J. Lukszo, K. Bialkowski and K. S. Kasprzak. *Chem. Res. Toxicol.* **11**, 1014 (1998).
10. W. Bal, R. Liang, J. Lukszo, S. H. Lee, M. Dizdaroglu and K. S. Kasprzak. *Chem. Res. Toxicol.* **13**, 616 (2000).
11. M. Mylonas, G. Malandrinos, J. C. Plakatouras, N. Hadjiliadis, K. S. Kasprzak, A. Krezel and W. Bal. *Chem. Res. Toxicol.* **14**, 1177 (2001).
12. M. Mylonas, J. C. Plakatouras, N. Hadjiliadis, A. Krezel and W. Bal. *Inorg. Chim. Acta.* **339**, 60 (2002).
13. M. Mylonas, A. Krezel, J. C. Plakatouras, N. Hadjiliadis and W. Bal. *J. Chem. Soc., Dalton Trans.* **2002**, 4296.
14. M. Mylonas, A. Krezel, J. C. Plakatouras, N. Hadjiliadis and W. Bal. In preparation.
15. H. Kozlowski, W. Bal, M. Dyba and T. Kowalik-Jankowska. *Coord. Chem. Rev.* **184**, 319 (1999).
16. H. Sigel and R. B. Martin. *Chem. Rev.* **82**, 385 (1982).
17. J. A. Tainer, E. D. Getzoff, J. S. Richardson and D. C. Richardson. *Nature.* **306**, 284 (1983).
18. D. T. Sawyer and J. S. Valentine. *Acc. Chem. Res.* **14**, 393 (1981).
19. J. Hirose and Y. Kidani, in: *Biocoordination Chemistry. Coordination Equilibria in Biologically Active Systems.* Ellis Horwood, Chichester, K. Burger Ed. 1990.
20. H. Irving, M. G. Miles and L. D. Pettit. *Anal. Chim. Acta.* **38**, 475 (1967).
21. P. Gans, A. Sabatini and A. Vacca. *J. Chem. Soc., Dalton Trans.* **1985**, 1195.
22. T. Kowalik-Jankowska, M. Ruta-Dolejsz, K. Wisniewska, L. Lankiewicz and H. Kozlowski. *J. Chem. Soc., Dalton Trans.* **2000**, 4511.
23. T. Kowalik-Jankowska, M. Ruta-Dolejsz, K. Wisniewska and L. Lankiewicz. *J. Inorg. Biochem.* **86**, 535 (2001).
24. M. Casolaro, M. Chelli, M. Ginanneschi, F. Laschi, L. Messori, M. Muniz-Miranda, A. M. Papini, T. Kowalik-Jankowska and H. kozlowski. *J. Inorg. Biochem.* **89**, 181 (2002).
25. R. P. Bonomo, F. Bonsignore, E. Conte, G. Impellizzeri, G. Pappalardo, R. Purrello and E. Rizzarelli. *J. Chem. Soc., Dalton Trans.* **1993**, 1295.
26. R. Vogler and H. Vahrenkamp. *Eur. J. Inorg. Chem.* **2002**, 761.
27. J. L.Yarger, R. A. Nieman and A. L. Bieber. *J. Chem. Educ.* **74**, 243 (1997).
28. R. S. Macomber. *J. Chem. Educ.* **69**, 375 (1992).
29. D. L. Rabenstein and T. L. Sayer. *Anal. Chem.* **48**, 1141 (1976).
30. E. Farkas, I. Sovago and A. Gergely. *J. Chem. Soc., Dalton Trans.* **1983**, 1545.

31. D. L. Rabenstein, S. A. Daignault, A. A. Isab, A. P. Arnold and M. M. Shoukry. *J. Am. Chem. Soc.* **107**, 6435 (1985).
32. T. Gajda, B. Henry and J. J. Delpuech. *J. Chem. Soc., Perkin Trans.* **1994**, 157.
33. R. Vogler and H. Vahrenkamp. *Eur. J. Inorg. Chem.* **2002**, 761.
34. P.Gockel, M. Gelinsky, R. Vogler and H. Vahrenkamp. *Inorg. Chim. Acta.* **272**, 115 (1998).
35. P. Gockel, R. Vogler, M. Gelinsky, A. Meibner, H. Albrich and H. Vahrenkamp. *Inorg. Chim. Acta.* **323**, 16 (2001).
36. S. A. Daignault, A. P. Arnold, A. A. Isab and D. L. Rabenstein. *Inorg. Chem.* **24**, 3984 (1985).
37. R. P. Agarwal and D. D. Perrin. *J. Chem. Soc., Dalton Trans.* **1975**, 1045.
38. P. Mlynarz, T. Kowalik-Jankowska, M. Stasiak, M. T. Leplawy and H. Kozlowski. *J. Chem. Soc., Dalton Trans.* **1999**, 3673.
39. J. -P. Laussac, H. Mazarguill, D. Prome, M. Erard and M. -T. Cung in *Genetic Response to Metals.* Marcel Dekker Inc, New York, B. Sarkar Ed., 1995.
40. A. Krezel, J. Wojcik, M. Maciejczyk and W. Bal. *Chem. Comm.* **2003**, 704.
41. E. C. Tibaduiza and D. J. Bobilya. *J. Cell. Physiol.* **167**, 539 (1996).

